# Supplementary material for: Machine learning prediction of combat basic training injury from 3D body shape images
Source: PLoS One. 2020 Jun 30;15(6):e0235017. doi: 10.1371/journal.pone.0235017 (PMC7326186; doi:10.1371/journal.pone.0235017)
Supplement: S1 Table — This is based on average across all cross-validation test folds using threshold yielding optimal TPR and FPR, and note this is slightly different than the TPR and FPR reported in the paper which is based on the optimal TPR/FPR of the average ROC curves. (DOCX) [file pone.0235017.s001.docx]

**Table S1.** Confusion matrix for the neural network model, with true positive rate (TPR) is 69.3%, and the false positive rate (FPR) is 35.2%. This is based on average across all cross-validation test folds using threshold yielding optimal TPR and FPR, and note this is slightly different than the TPR and FPR reported in the paper which is based on the optimal TPR/FPR of the average ROC curves.

|  |  | Predicted | |
| --- | --- | --- | --- |
|  |  | Non-injured | Injured |
| Actual | Non-injured | 2,817 | 1,533 |
|  | Injured | 10 | 23 |
